# 2021 SAEM Consensus Conference Proceedings: Research Priorities for Developing Emergency Department Screening Tools for Social Risks and Needs

**DOI:** 10.5811/westjem.2022.8.57271

**Published:** 2022-10-10

**Authors:** Jacqueline Furbacher, Callan Fockele, Ben Del Buono, Laura Janneck, Cooper March, Melanie Molina, Herbet C Duber, Kelly M Doran, Michelle P Lin, Richelle J Cooper, Payal Modi

**Affiliations:** * University of Massachusetts Chan Medical School, Department of Emergency Medicine, Worcester, Massachusetts; † University of Washington, Department of Emergency Medicine, Seattle, Washington; ‡ Virginia Commonwealth University, Department of Emergency Medicine, Richmond, Virginia; § University of Oklahoma School of Community Medicine, Department of Emergency Medicine, Tulsa, Oklahoma; ¶ Stanford University School of Medicine, Department of Emergency Medicine, Stanford, California; || University of California, San Francisco, Department of Emergency Medicine, San Francisco, California; # NYU School of Medicine, Departments of Emergency Medicine and Population Health, New York, New York; ** UCLA School of Medicine, Department of Emergency Medicine, Los Angeles, California

## Abstract

**Introduction:**

The Emergency Department (ED) acts as a safety net for our healthcare system. While studies have shown increased prevalence of social risks and needs among ED patients, there are many outstanding questions about the validity and use of social risks and needs screening tools in the ED setting.

**Methods:**

In this paper, we present research gaps and priorities pertaining to social risks and needs screening tools used in the ED, identified through a consensus approach informed by literature review and external expert feedback as part of the 2021 SAEM Consensus Conference -- From Bedside to Policy: Advancing Social Emergency Medicine and Population Health.

**Results:**

Four overarching research gaps were identified: (1) Defining the purpose and ethical implications of ED-based screening; (2) Identifying domains of social risks and needs; (3) Developing and validating screening tools; and (4) Defining the patient population and type of screening performed. Furthermore, the following research questions were determined to be of highest priority: (1) What screening tools should be used to identify social risks and needs? (2) Should individual EDs use a national standard screening tools or customized screening tools? (3) What are the most prevalent social risks and needs in the ED? and (4) Which social risks and needs are most amenable to intervention in the ED setting?

**Conclusion:**

Answering these research questions will facilitate the use of evidence-based social risks and needs screening tools that address knowledge gaps and improve the health of our communities by better understanding the underlying determinants contributing to their presentation and health outcomes.

## INTRODUCTION

The World Health Organization defines social determinants of health (SDoH) as “conditions in which people are born, grow, live, work and age…[which are] shaped by the distribution of money, power and resources at global, national and local levels.”^1^ The SDoH affect health outcomes,^2^ health system costs and healthcare utilization for all populations along the spectrum of health and wellbeing.^3^,^4^ Some people have used the term “social determinants of health” interchangeably with “social needs” and “social risk factors.” Alderwick and Gottlieb clarified terminology related to SDoH to standardize language and facilitate national discussion of practices related to SDoH in healthcare. Whereas *social risk* encompasses “specific adverse social conditions that are associated with poor health, such as social isolation or housing instability,” *social need* also incorporates consideration of patients’ “preferences and priorities” for assistance.^4^ Social risks and needs focus on the individual, while SDoH take a broader view of the underlying structural and environmental factors contributing to health.^4^ Identifying individual social risks and needs provides an opportunity to promote interventions to directly address the social risks and needs and their subsequent contribution to health.^4^

Current literature on screening for social risks and needs focuses primarily on the outpatient clinical setting.^5^,^6^ However, the ED offers a unique opportunity to identify individuals with social risks and needs given its role as a safety net in the US healthcare system. Additionally, patients with increased social risks and needs are more apt to use the ED.^7^–^10^ An evidenced based screening process for social risks and needs in ED populations is yet to be defined, validated, and widely accepted in routine practice. As a result, we reviewed relevant literature to explore existing ED social risks and needs screening tools, identify gaps in the literature, and propose future research priorities. This work was presented to consensus conference attendees meeting virtually during the April 2021 Society for Academic Emergency Medicine (SAEM) Consensus Conference—From Bedside to Policy: Advancing Social Emergency Medicine and Population Health through Research, Collaboration, and Education. The two-part Consensus Conference concluded with a final, revised list of research priorities.

This manuscript is the first of three addressing various aspects of the continuum from screening to interventions for social risks and needs in the ED setting. Here, we review current literature pertaining to the development and validity of instruments used for social risks and needs screening, and present research priorities derived through a consensus process.

## METHODS

The leadership team of the SAEM Consensus Conference session on social risks and needs screening identified three topics for review: 1) instruments used for social risks and needs screening in the ED; (2) implementation of social risks and needs screening in the ED; and (3) interventions for patients with social risks and needs in the ED.^11^ Each of these topics was assigned to a workgroup led by two individuals, at least one of whom had significant experience in the field of social risks and needs. Emergency physicians, residents, and medical students were recruited through an open call to join, and subsequently assigned to one of the three research workgroups. The leadership team members supported all three groups. This manuscript addresses the first topic, presenting a review of existing literature for social risk and needs screening instruments and associated consensus-based research priorities.

### Literature Review

We conducted a literature review, adapting methodology from a published systematic review on ED patients’ social needs.^12^ With the assistance of a health sciences librarian, we used a PubMed search strategy (Appendix 1) to identify 2,085 articles across the continuum of social risks and needs screening. Titles and abstracts were screened, resulting in 151 potentially relevant manuscripts. This initial search was complemented with a review of the Social Interventions Research & Evaluation Network (SIREN) Evidence and Resource Library,^13^ which compiles research on medical and social care integration. This resulted in 22 additional articles for review. Of the 173 total manuscripts identified, 92 were deemed potentially relevant to the topic of instruments used for screening of social risks and needs in the ED. The PubMed and SIREN database searches were conducted in December 2020.

A member of our workgroup reviewed each of these 92 publications, extracting information pertaining to study objective, design, outcomes, results, limitations, and quality into a database. The literature review focused on examining what screening instruments were used, how they were derived and validated, and what content they covered. Finally, the workgroup performed a supplemental literature search of the bibliographic references in the included articles to identify additional relevant studies. Thirteen additional articles were identified and reviewed using the same process described above. We included a total of 105 articles in our final assessment (Figure). Pertinent data was extracted from each manuscript and included in a Microsoft Excel for Mac file, version 16.52 (Microsoft Corporation, Redmond, WA) database.

### Initial Derivation of Research Gaps and Priorities

The workgroup used an iterative consensus process to derive research gaps and draft preliminary research priorities based on the information included in the literature review database. Domains are categories of social risks and needs as described by the US Department of Health and Human Services (HHS).^14^ They include economic stability, education access and quality, healthcare access and quality, neighborhood and built environment, and social and community context.^14^ Within these larger domains are employment, housing, literacy, language, access to healthy food, exposure to violence, and more. We chose this framework of domains to better understand the breadth of literature reviewed on social risks and needs screening. Furthermore, this helped clarify social risks and needs that are understudied in the ED. The workgroup then shared a list of draft research priorities with external experts from the HHS Office of the Assistant Secretary for Planning and Evaluation,^15^ Health Leads,^16^ and SIREN.^17^ Feedback was solicited from these external experts and integrated into a prereading document of preliminary research priorities shared with SAEM Consensus Conference participants.

### Consensus Building and Derivation of Final Research Gaps and Priorities

The Consensus Conference occurred over two virtual meetings via Zoom on April 13 and April 27, 2021, during the SAEM Consensus Conference. Consensus was reached through a stepwise process, beginning with a presentation of methods used in the literature review and process of developing preliminary research priorities. A moderated discussion followed, allowing for all participants to provide verbal feedback. Between the first and second meetings, preliminary research priorities were sent to participants to solicit additional comment and ranking of priorities with an electronic survey that asked conference attendees the following questions:

1. Are there any research priorities that you feel are missing from this list? Yes/No
  - If yes, please list them and note why they should be added.
2. Are there any research priorities that you feel should be removed? Yes/No
  - If yes, please list them and note why they should be removed.
3. Which research priorities should be discussed further in the April 27 breakout sessions? Why?
4. Please rank the top three research priorities based upon their priority for future research. Please consider the SMART criteria (specific, measurable, attainable, relevant, time-based) when completing this exercise.

Our workgroup then incorporated feedback from discussion during the first session and intersession survey, modifying research priorities into a revised list of research priorities. The second Consensus Conference session on April 27 focused on this revised list of priorities, with special attention paid to those that ranked lowest in the intersession survey. Minor changes were made as the group moved toward consensus, resulting in a final list of research priorities. This list was then sent to all Consensus Conference attendees who participated in any part of the ED screening sessions, and they were asked to rank the final priorities list based on the SMART criteria. Research priorities were scored using the following formula:


$$\text{Total score}=3\times (\#\text{of }1^{\text{st}}\text{-choice votes})+2\times (\#\text{of }2^{\text{nd}}\text{-choice votes})+1\times (\#3^{\text{rd}}\text{-choice votes})$$

This resulted in a final list of ranked research priorities—high, medium, or low priority—based on relative score (top ⅓, middle ⅓, lowest ⅓, respectively). Below, we present research priorities pertaining to social risks and needs screening instruments grouped by key thematic gaps in the literature. See the table for final ranked research priorities pertaining to instruments used for social risks and needs screening in the ED.

## FINDINGS and DISCUSSION

The working group reviewed 105 articles pertinent to social risks and needs screening in the ED. A wide range of social risks and needs were addressed in the studies. Some focused on specific social risks and needs while others looked at a general grouping of social risks and needs.^18^–^43^ Articles were sorted by general domains from the HHS framework to provide a broad understanding of gaps in specific social needs and risks screening tools.^14^ Specific aims within various domains included developing ED-specific screening tools, validating screening tools, understanding the accuracy of screening tools, and understanding the prevalence of social risks and needs in a specific ED setting. This initial analysis prompted robust discussion on gaps and priorities related to social risks and needs screening.

### Gap 1: Defining the purpose and ethical implications of ED-based screening

During the Consensus Conference, conversations about social risks and needs shifted to the ethics of ED-based screening. Participants expressed concern about identifying patients to screen and the potential for stigma associated with it. Additionally, patient perception of screening could impact screening success and the patient-physician relationship. For example, the identification of social risks that patients do not perceive as social needs may be perceived as intrusive if unrelated to patients’ presenting issues. Further understanding of ED patient perception regarding social risks and needs screening is necessary. Participants also discussed the ethical implications of screening for social risks and needs without clear interventions or solutions. For example, screening is necessary to measure the prevalence of social risks and needs in ED populations, which is a prerequisite to obtaining resources and developing new interventions; however, interventions may not yet exist to address identified risks and needs at the time of screening. Consistent language regarding screening purposes and uses may alleviate these concerns and requires further study.

#### Research Priorities

1. Should EDs screen patients for social risks, social needs, or both?
2. What are the ethical boundaries of implementing screening tools in the ED?

### Gap 2: Domains of social risks and needs

The range and types of social domains screened for varied among studies.^18^–^43^ Some literature focused on multiple domains while others looked primarily at a single social risk or need such as food insecurity or intimate partner violence (IPV).^18^–^43^ Optimizing social domains is an important step when evaluating ED screening tools. While there is no established set of domains for ED-based social risks and needs screening, examples exist in other screening frameworks. For example, the Accountable Health Communities model, a nationwide screening tool that addresses health-related social risks and needs among Medicare and Medicaid beneficiaries, established five core domains for screening questions: living situation; food; transportation; utilities; and safety.^44^ Other models, such as the National Association of Community Health Centers Protocol for Responding to and Assessing Patients’ Assets, Risks, and Experiences (PRAPARE), describe core measures informed by SDoH domains, including personal characteristics, family and home, money and resources, social and emotional health, and safety.^45^

Existing literature on ED screening and screening tools is heavily weighted toward certain domains. There are multiple studies examining IPV, substance use disorder, and mental health in ED populations using validated screening tools.^46^–^54^ Additionally, food insecurity and housing/homelessness were commonly screened for in both multi-domain screener studies as well as in isolation.^18^,^23^,^27^,^29^,^34^,^35^,^38^,^39^,^55^–^69^ Transportation access was included in multi-domain screening; however, it has yet to be studied in isolation.^6^,^20^,^23^,^28^,^33^–^36^,^38^,^41^–^43^ Emergency department screening of violence focused on IPV or domestic violence; there are fewer studies regarding ED screening for elder abuse, child abuse, exposure to violence, or human trafficking.^46^–^54^,^70^–^108^ Significant gaps in the ED-based literature on social risks and needs were found for domains such as neighborhood conditions and health literacy. There is no consensus in the literature regarding methods or criteria to determine domains of social risks and needs pertinent to ED screening generally or within a specific ED setting. There was discussion in the Consensus Conference about how geographic location may be an important factor in determining which domains are relevant for screening.

#### Research Priorities

1. Which domains of social risks and needs are considered most pertinent to social emergency medicine? (Housing, IPV, food insecurity, etc)
2. What theoretical model (eg, Maslow’s hierarchy of needs) should we apply to better understand domains of social risks and needs?
3. Which domains of social risks and needs are most prevalent among ED patients, have the largest impact on health, and are most amenable to ED-based screening and interventions?

### Gap 3: Development and validation of screening tools

Our literature review noted many screening tools for social risks and needs in the ED population lack robust validation. This is particularly evident for screening tools identifying multiple social risks and needs. The Hunger Vital Sign is validated and widely accepted as a screening tool for food insecurity in the ED.^56^–^57^ Other validated screening tools have been employed in screening for domestic violence, substance use, and mental health including anxiety, depression, post-traumatic stress disorder, and stress.^46^–^54^,^81^–^108^ A brief validated screening tool does not exist for evaluating housing insecurity or multiple social risks and needs. Both topics were common themes in the literature despite the lack of validated screening tools.^18^–^43^,^63^–^69^ In studies that developed screening tools or developed their own screening questions, internal validation techniques such as cognitive interviews with sample populations were used.^18^,^19^,^23^,^24^, ^38^,^63^,^73^,^79^,^103^ The reliability and validity of these tools for general use in ED populations is unknown.

Further, there are often instances where multiple screening tools exist for the same social risks and needs. For example, the Partner Violence Screen, Revised Conflict Tactic Scale, and AUDIT-C were all used to identify domestic violence.^46^–^54^,^81^–^107^ Different instruments for the same risks and needs are rarely compared to one another. This makes comparisons between populations difficult and creates challenges interpreting the utility of interventions based on positive responses to different screening tools.

Consensus Conference participants recognized the importance of rigorous screening tool development and validation. However, many challenges exist to the implementation of such instruments. Rigorous development using cognitive interviews, and internal and external validation is time-consuming and resource intensive. It was agreed that community partners, patients, and other key stakeholders should be engaged in the development of screening tools and questions. This ensures broader buy-in and prevents unintended consequences that may arise from asking highly sensitive questions to vulnerable communities. The literature primarily focused on screening in English-speaking patients. Few studies screened patients using other languages; among the minority that engage non-English speakers, most used Spanish. Extensive gaps exist with regard to language translation and tailoring screening questions by language.^23^,^31^,^35^,^40^,^49^,^50^,^61^,^65^,^84^,^106^,^114^,^120^ Limited studies examined screening tools at multiple EDs or across geographic regions.^21^,^30^,^34^,^62^,^74^,^99^,^87^,^107^ Consensus Conference participants advocated for development and validation of standardized screening tools to allow for data collection and comparisons nationally and to advance the field.

#### Research Priorities

1. What screening tools should we be using to screen for social risks and/or social needs in the ED? Is there a benefit to using standardized tools across all EDs nationally? To what extent should EDs customize their own instruments (eg, for various geographic settings)?
2. Do existing screening questions and tools need to be validated in the ED setting, or is it sufficient if they have been validated in other settings? Do screening tools that have been modified for ED use perform similarly to originally validated screening tools?
3. What is the impact of language translation on screening tool performance?
4. How do we incorporate community partners, patients, and key stakeholders in developing or modifying existing screening tools?

### Gap 4: Defining the patient population and type of screening performed

Comprehensive screening addresses all social risks and needs, while focused screening only includes certain social risks and needs thought to be relevant to the respective patient population. Both strategies are found in the existing ED literature, but insufficient research exists to determine which approach is most successful and pertinent to the ED.^27^,^30^,^43^,^45^,^46^,^48^–^52^ The most critical difference between these strategies is the time it takes to perform a more comprehensive screening. Conference participants proposed using a brief, comprehensive screening strategy to identify social risks and needs pertinent to the specific ED population and to use it for focused screening. However, it was also acknowledged that this may create a false hierarchy of importance among social risks and needs and result in important issues going unaddressed during an ED encounter.

Universal screening is the process of screening all patients within a hospital or health system for social risks and needs, while targeted screening involves approaching only a selected subset of patients based on perceived risk or need (eg, age-based screening for elder abuse). Discussion of who is approached for screening in the ED and what social risks and needs are addressed was prevalent at the Consensus Conference. Proponents for universal screening noted this approach promotes equity and limits implicit bias. However, it was generally acknowledged that time and resource constraints in the ED setting are important considerations.

#### Research Priorities

1. Are there social risks and needs that should be screened for universally in all ED patients across the United States?

## CONCLUSION

There is a growing body of research on instruments used for screening for social risks and needs in the ED setting; however, many unanswered questions remain. Key topics include the use of a common language/framework when assessing social risks and needs, as well as establishing a theoretical model to frame the research on screening and intervening for social risks and needs in the ED. Further, defining domains to be included in ED-based screening, developing validated instruments in multiple languages, and clarifying how different instruments can be used and compared to one another will help fill in important gaps in our current knowledge. Expanding research to ensure the use of validated tools for social risks and needs screening in the ED has the potential to promote data-driven healthcare policy that serves to improve health disparities. Emergency department-based screening represents an opportunity to reach marginalized populations that may not present to other healthcare environments. Research gaps and priorities identified through the consensus process offer direction for future studies to establish validated screening methods and/or best practices for identifying social risks and needs in ED populations.

## Supplementary Information





## Figures and Tables

**Figure:**
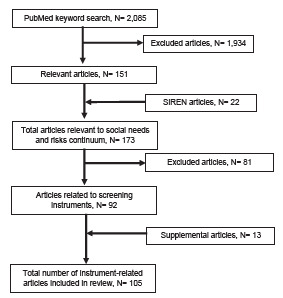
Flow diagram of literature review search results.

**Table 1:** Final ranked research priorities pertaining to instruments used for social risks and needs screening in the emergency department. Total score is weighted (3 points for priority 1 vote, 2 points for priority 2 vote, and 1 point for priority 3 vote).

Research questions	Priority 1	Priority 2	Priority 3	Total points	Priority
Which domains of social risks and needs (eg, housing, interpersonal violence, and food insecurity) are considered most pertinent to social emergency medicine? Which domains of social risks and needs are most prevalent among ED patients, have the largest impact on health, and are most amenable to ED-based screening and interventions?	15	7	3	62	High
What screening tools should we be using to screen for social risks and needs in the ED? Is there a benefit to using standardized tools across all EDs nationally? To what extent should EDs customize their own instruments (eg, for various geographic settings)?	6	12	7	49	High
Should EDs screen patients for social risks, social needs, or both? What are the ethical boundaries of implementing screening tools in emergency medicine?	6	5	2	30	Medium
What is the impact of language translation on screening tool performances? How do we incorporate community partners, patients, and key stakeholders in developing or modifying existing screening tools?	3	5	5	24	Medium
Do existing screening questions and tools need to be validated in the ED setting, or is it sufficient if they have been validated in other settings? Do screening tools that have been modified for ED use perform similarly to originally validated screening tools?	0	4	12	20	Medium
Are there social risks and needs that should be screened for universally in all ED patients across the country?	4	1	3	17	Low
What theoretical models (eg, Maslow’s hierarchy of needs) should we apply to better understand domains of social risks and needs?	0	0	2	2	Low

*ED*, emergency department.
