# Supplementary material for: 2021 SAEM Consensus Conference Proceedings: Research Priorities for Developing Emergency Department Screening Tools for Social Risks and Needs
Source: West J Emerg Med. 2022 Oct 10;23(6):817–22. doi: 10.5811/westjem.2022.8.57271 (PMC9683763; doi:10.5811/westjem.2022.8.57271)
Supplement: Supplementary file 1 [file wjem-23-817-s001.docx]

**Appendix A**: Literature Review Search Terms

|  |  |
| --- | --- |
| SDOH general | ("socioeconomic factors" [Majr:noexp]  “social conditions”[Majr]  "social change" [Majr]  "social class" [Majr]  "social environment" [Majr]  "family characteristics" [Majr]  "Social Determinants of Health" [Mesh]  "Social Environment" [Mesh]  "Health Status Disparities" [Mesh]  "social needs" [tiab]  social determinant*[tiab]  "material needs" [tiab]  social deprivation*[tiab]  social risk*[tiab]  structural vulnerability[tiab]) |
| built environment | ("Built Environment"[Mesh]  built environment[tiab]) |
| childcare | ("Child Care"[Mesh]  Childcare[tiab]  Child care[tiab]  Daycare[tiab]) |
| criminal justice | (Criminal Justice[tiab]  justice involved[tiab]  justice system involved[tiab]  criminal record*[tiab]  criminal history[tiab]  prison[tiab]) |
| discrimination | ("Social Discrimination"[MeSH Terms]  "Prejudice"[MeSH Terms]  "Social Marginalization"[MeSH Terms]  "Social Stigma"[MeSH Terms]  "marginalization"[Title/Abstract]  "Homophobia"[Title/Abstract]  "Racism"[Title/Abstract]  "Sexism"[Title/Abstract]  "race bias"[Title/Abstract]  "racial bias"[Title/Abstract]  "implicit bias"[Title/Abstract]  "explicit bias"[Title/Abstract]  "covert bias"[Title/Abstract]  "ethnic bias"[Title/Abstract]  "gender bias"[Title/Abstract]  "sex bias"[Title/Abstract]  "age bias"[Title/Abstract]  "religious bias"[Title/Abstract]  "weight bias"[Title/Abstract]  "race discrimination"[Title/Abstract]  "racial discrimination"[Title/Abstract]  "ethnic discrimination"[Title/Abstract]  "gender discrimination"[Title/Abstract]  "sex discrimination"[Title/Abstract]  "age discrimination"[Title/Abstract]  "religious discrimination"[Title/Abstract]  "sexuality discrimination"[Title/Abstract]  "weight discrimination"[Title/Abstract]) |
| economic security | (income [majr:noexp]  poverty [majr]  medical indigency [majr]  "Working Poor"[Mesh]  low income[tiab]  financial toxicity[tiab]  financial hardship[tiab]) |
| education/literacy | ("Literacy"[Mesh]  "Educational Status"[Mesh]  "Academic Failure"[Mesh]  Literacy[tiab]  Illiteracy[tiab]  Educational achievement*[tiab]  Educational status[tiab]  Academic failure*[tiab]  Academic success*[tiab]  reading level[tiab]) |
| employment | (employment [Majr]  unemployment [Majr]  return to work [Majr]  Precarious Employment[tiab]  Marginal Employment[tiab]  Employment Insecurit*[tiab]  underemploy*[tiab]  unemploy*[tiab]) |
| food/hunger | (hunger [majr:noexp]  malnutrition [tiab]  malnourished [tiab]  food assistance [majr]  food insecur*[tiab]  "hunger"[tiab]  “Malnutrition"[Mesh]  "Food Assistance"[Mesh]  "Food Supply"[Mesh]  “Supplemental Nutrition Assistance Program”[tiab]  “Women, Infants, and Children Program”[tiab]  Food Stamp*[tiab]  Nutritional Deficienc*[tiab]  Undernutrition[tiab]  Malnourishment[tiab]  Food Security[tiab]  Food scarcity[tiab]  Food aid[tiab]  Food pantr*[tiab]  food bank*[tiab]  Emergency food[tiab]  Food shelf[tiab]) |
| health care access | (("Medical Assistance"[Mesh] AND (social determinant*[tiab] OR "Social Determinants of Health"[Mesh]))  "Medically Uninsured" [Mesh]  "Health Services Accessibility" [Mesh]  "Health services availability" [tiab]  "health care availability" [tiab]  "healthcare availability" [tiab]  "Health services accessibility" [tiab]  "health care accessibility" [tiab]  "healthcare accessibility" [tiab]  "Health services access" [tiab]  "health care access" [tiab]  "healthcare access" [tiab]  "access to Health services" [tiab]  "access to health care" [tiab]  "access to healthcare" [tiab]  "Healthcare Disparities" [Mesh]  Health Care Inequalit*[tiab]  Healthcare inequalit*[tiab]  Health Care Disparit*[tiab]  Healthcare Disparit*[tiab]  Medicaid[tiab]  Uninsured[tiab]) |
| housing quality | (housing [majr:noexp]  public housing [majr]  "housing" [tiab]  "Residence Characteristics"[Mesh]) |
| housing stability | (homeless persons [Majr]  homeless youth [Majr]  "homeless"[tiab]  Homelessness[tiab]  housing stability[tiab]  housing instability[tiab]  Unhoused[tiab]  stable housing[tiab]  unstable housing[tiab]  housing status[tiab]  Evict*[tiab]  Shelter[tiab]  Unsheltered[tiab]) |
| immigration | ("Emigration and Immigration"[Mesh]  "Emigrants and Immigrants"[Mesh]  "Transients and Migrants"[Mesh]  immigration status[tiab]  undocumented[tiab]  Immigrant*[tiab]  Citizenship[tiab]  Migrant*[tiab]) |
| legal services | ("Legal Services"[Mesh] OR legal services[tiab] OR legal representation[tiab] OR Legal advoca*[tiab]) |
| public benefits | ("Public Assistance"[Mesh:noexp]  "Old Age Assistance"[Mesh]  "Social Security"[Mesh]  SSI[tiab]  public aid[tiab]  public assistance[tiab]  public benefits[tiab]  government aid[tiab]  government assistance[tiab]  government benefits[tiab]  Safety net program*[tiab]  social safety net[tiab]) |
| social support/social isolation | ("Social Support"[Mesh]  "Community Networks"[Mesh]  "Social Networking"[Mesh]  "Social Isolation"[Mesh]  "Homebound Persons"[Mesh]  social support[tiab]  community network*[tiab]  social network*[tiab]  social isolation[tiab]  socially isolated[tiab]  Social connection*[tiab]  Homebound[tiab]  Home-bound[tiab]  Housebound[tiab]  House-bound[tiab]  Shut-in*[tiab]  social vulnerability[tiab]) |
| transportation | (((lack*[tiab] OR availa*[tiab] OR access*[tiab] OR obtain*[tiab]) AND transportation[tiab]) OR Car ownership[tiab]) |
| utilities | ((Utilities[tiab] OR Water[tiab] OR Electricity[tiab] OR Heat*[tiab] OR Fuel[tiab] OR gas[tiab])  AND  (insecurity[tiab] OR “cut off” [tiab] OR “turned off” [tiab] OR Disconnect*[tiab] OR Nonpayment[tiab] OR Problems[tiab] OR assistance[tiab])) |
| violence/safety | ((“Violence”[Mesh]  Domestic violence[tiab]  family violence[tiab]  Intimate partner violence[tiab]  Intimate partner abuse[tiab]  Child abuse[tiab]  Child mistreatment[tiab]  Child maltreatment[tiab]  Child neglect[tiab]  Elder abuse[tiab]  Elder neglect[tiab]  Aged abuse[tiab]  spousal abuse[tiab]  partner abuse[tiab]  wife abuse[tiab]  husband abuse[tiab]  physical abuse[tiab]  gun violence[tiab]  physical violence[tiab]  physical abuse[tiab]  Assault*[tiab]  Personal safety[tiab]  Unsafe[tiab]  victim[tiab])  NOT "Workplace Violence" [Mesh] NOT "Workplace Violence") |
