# Supplementary material for: 2021 SAEM Consensus Conference Proceedings: Research Priorities for Developing Emergency Department Screening Tools for Social Risks and Needs
Source: West J Emerg Med. 2022 Oct 10;23(6):817–22. doi: 10.5811/westjem.2022.8.57271 (PMC9683763; doi:10.5811/westjem.2022.8.57271)
Supplement: Supplementary file 2 [file wjem-23-817-s002.docx]

**REFERENCES**

1. WHO | About social determinants of health. WHO. Accessed October 9, 2019. http://www.who.int/social_determinants/sdh_definition/en/
2. Samantha Artiga, Elizabeth Hinton. *Beyond Health Care: The Role of Social Determinants in Promoting Health and Health Equity.* Henry J. Kaiser Family Foundation; 2018.
3. Social Determinants of Health - Healthy People 2030 | health.gov. Accessed June 11, 2021. https://health.gov/healthypeople/objectives-and-data/social-determinants-health
4. Alderwick H, Gottlieb LM. Meanings and Misunderstandings: A Social Determinants of Health Lexicon for Health Care Systems. *Milbank Q*. 2019;97(2):407-419.
5. Beck AF, Cohen AJ, Colvin JD, et al. Perspectives from the Society for Pediatric Research: interventions targeting social needs in pediatric clinical care. *Pediatr Res*. 2018;84(1):10-21.
6. Gottlieb LM, Hessler D, Long D, et al. Effects of Social Needs Screening and In-Person Service Navigation on Child Health: A Randomized Clinical Trial. *JAMA Pediatr*. 2016;170(11):e162521.
7. Cole MB, Nguyen KH. Unmet social needs among low‐income adults in the United States: Associations with health care access and quality. *Health Serv Res*. 2020;55(S2):873-882.
8. Kubín V, Franĕk J. [Frequency of excretion of group B streptococci (Streptococcus agalactiae) in the milk during subclinical forms of mammary gland diseases in cows]. *Vet Med (Praha)*. 1984;29(3):129-132.
9. McQueen A, Li L, Herrick CJ, et al. Social Needs, Chronic Conditions, and Health Care Utilization among Medicaid Beneficiaries. *Popul Health Manag*. Published online May 14, 2021:pop.2021.0065
10. Mosen DM, Banegas MP, Benuzillo JG, et. al. Association Between Social and Economic Needs With Future Healthcare Utilization. *Am J Prev Med*. 2020;58(3):457-460.
11. Schoenfeld EM, Lin MP, Samuels-Kalow ME. Executive Summary of the 2021 SAEM Consensus Conference: From Bedside to Policy: Advancing Social Emergency Medicine and Population Health Through Research, Collaboration, and Education [published online ahead of print, 2022 Jan 22]. *Acad Emerg Med*. 2022;10.1111/acem.14451.
12. Malecha PW, Williams JH, Kunzler NM, Goldfrank LR, Alter HJ, Doran KM. Material Needs of Emergency Department Patients: A Systematic Review. Asher SL, ed. *Acad Emerg Med*. 2018;25(3):330-359.
13. Social Interventions Research & Evaluation Network (SIREN). Evidence and Resource Library. University of San Francisco, CA. https://sirenetwork.ucsf.edu/tools/evidence-library. Accessed 2021.
14. U.S. Department of Health and Human Services; Office of Disease Prevention and Health Promotion. (n.d.). *Social Determinants of Health*. Social Determinants of Health | Healthy People 2020. Accessed March 13, 2022. https://www.healthypeople.gov/2020/topics-objectives/topic/social-determinants-of-health
15. Office of the Assistant Secretary for Planning and Evaluation (ASPE). U.S. Department of Health and Human Services. https://aspe.hhs.gov. Accessed 2021.
16. Health leads. https://healthleadsusa.org. Accessed 2021.
17. Social Interventions Research & Evaluation Network (SIREN). University of San Francisco, CA. https://sirenetwork.ucsf.edu. Accessed 2021.
18. Bisgaier J, Rhodes KV. Cumulative Adverse Financial Circumstances: Associations with Patient Health Status and Behaviors. *Health Soc Work*. 2011;36(2):129-137.
19. Bourgois P, Holmes SM, Sue K, Quesada J. Structural Vulnerability: Operationalizing the Concept to Address Health Disparities in Clinical Care. *Acad Med*. 2017;92(3):299-307.
20. Burks CE, Jones CW, Braz VA, et al. Risk Factors for Malnutrition among Older Adults in the Emergency Department: A Multicenter Study. *J Am Geriatr Soc*. 2017;65(8):1741-1747.
21. Caitlin Thomas-Henkel, Meryl Schulman. *Screening for Social Determinants of Health in Populations with Complex Needs: Implementation Considerations*. Center for Health Care Strategies, Inc.; 2017. https://www.chcs.org/media/SDOH-Complex-Care-Screening-Brief-102617.pdf
22. Cartier Y, Fichtenberg C, Gottlieb LM. Implementing Community Resource Referral Technology: Facilitators And Barriers Described By Early Adopters: A review of new technology platforms to facilitate referrals from health care organizations to social service organizations. *Health Aff*. 2020;39(4):662-669.
23. Ciccolo G, Curt A, Camargo Jr. C, Samuels-Kalow M. Improving Understanding of Screening Questions for Social Risk and Social Need Among Emergency Department Patients. *West J Emerg Med*. 2020;21(5).
24. Cook JT, Frank DA, Casey PH, et al. A Brief Indicator of Household Energy Security: Associations With Food Security, Child Health, and Child Development in US Infants and Toddlers. *Pediatrics*. 2008;122(4):e867-e875.
25. Fichtenberg CM, Alley DE, Mistry KB. Improving Social Needs Intervention Research: Key Questions for Advancing the Field. *Am J Prev Med*. 2019;57(6):S47-S54.
26. Gerber E, Gelberg L, Rotrosen J, Castelblanco D, Mijanovich T, Doran KM. Health-related material needs and substance use among emergency department patients. *Subst Abus*. 2020;41(2):196-202.
27. Gordon JA. Where health and welfare meet: social deprivation among patients in the emergency department. *J Urban Health.* 2001;78(1):104-111.
28. Gottlieb L, Hessler D, Long D, Amaya A, Adler N. A Randomized Trial on Screening for Social Determinants of Health: the iScreen Study. *Pediatrics*. 2014;134(6):e1611-e1618.
29. Gottlieb L, Hessler D, Long D, Amaya A, Adler N. A Randomized Trial on Screening for Social Determinants of Health: the iScreen Study. *Pediatrics*. 2014;134(6):e1611-e1618.
30. Gottlieb L, Hessler D, Long D, et al. Are acute care settings amenable to addressing patient social needs: A sub-group analysis. *Am J Emerg Med*. 2018;36(11):2108-2109.
31. Gottlieb LM, Adler NE, Wing H, et al. Effects of In-Person Assistance vs Personalized Written Resources About Social Services on Household Social Risks and Child and Caregiver Health: A Randomized Clinical Trial. *JAMA Netw Open*. 2020;3(3):e200701.
32. Gottlieb LM, Wing H, Adler NE. A Systematic Review of Interventions on Patients’ Social and Economic Needs. *Am J Prev Med*. 2017;53(5):719-729.
33. Jackson TS, Moran TP, Lin J, Ackerman J, Salhi BA. Homelessness Among Patients in a Southeastern Safety Net Emergency Department. *South Med J.* 2019;112(9):476-482.
34. Kandasamy D, Platts-Mills T, Shah M, Van Orden K, Betz M. Social Disconnection Among Older Adults Receiving Care in the Emergency Department. *W J Emerg Med*. 2018;19(6):919-925.
35. Malecha PW, Williams JH, Kunzler NM, Goldfrank LR, Alter HJ, Doran KM. Material Needs of Emergency Department Patients: A Systematic Review. Asher SL, ed. *Acad Emerg Med*. 2018;25(3):330-359. doi:10.1111/acem.13370
36. Molina M, Li C, Manchanda E, et al. Prevalence of Emergency Department Social Risk and Social Needs. *West J Emerg Med*. 2020;21(6). doi:10.5811/westjem.2020.7.47796
37. O’Gurek DT, Henke C. A Practical Approach to Screening for Social Determinants of Health. *Fam Pract Manag*. 2018;25(3):7-12.
38. Quigg AM. WIC Participation and Attenuation of Stress-Related Child Health Risks of Household Food Insecurity and Caregiver Depressive Symptoms. *Arch Pediatr Adolesc Med*. 2012;166(5):444.
39. Reves SR, O’Neal JP, Gonzalez MM, McHenry C, Favour M, Etz RS. A 60-Second Survey to Identify Patients’ Unmet Social Needs. *Ann Fam Med*. 2019;17(3):274-274.
40. Rodriguez RM, Fortman J, Chee C, Ng V, Poon D. Food, Shelter and Safety Needs Motivating Homeless Persons’ Visits to an Urban Emergency Department. *Ann Emerg Med*. 2009;53(5):598-602.e1.
41. Semple-Hess JE, Pham PK, Cohen SA, Liberman DB. Community Resource Needs Assessment Among Families Presenting to a Pediatric Emergency Department. *Acad Pediatr*. 2019;19(4):378-385.
42. Smith SK, Johnston J, Rutherford C, Hollowell R, Tanabe P. Identifying Social-Behavioral Health Needs of Adults with Sickle Cell Disease in the Emergency Department. *J Emerg Nurs*. 2017;43(5):444-450.
43. Stevens TB, Richmond NL, Pereira GF, Shenvi CL, Platts-Mills TF. Prevalence of Nonmedical Problems Among Older Adults Presenting to the Emergency Department. Gerson L, ed. *Acad Emerg Med*. 2014;21(6):651-658.
44. Zambrana RE, Ell K, Dorrington C, Wachsman L, Hodge D. The Relationship between Psychosocial Status of Immigrant Latino mothers and Use of Emergency Pediatric Services. *Health Soc Work*. 1994;19(2):93-102.
45. Billioux A, Verlander K, Anthony S, et al. Standardized screening for health-related social needs in clinical settings: the accountable health communities screening tool. *NAM Perspect.* 2017. https://nam.edu/standardized-screening-for-health-related-social-needs-in-clinical-settings-the-accountable-health-communities-screening-tool/
46. About the PRAPARE Assessment Tool. National Association of Community Health Centers. Accessed November 5, 2021. http://prapare.org/what-is-prapare/
47. Caetano R, Cunradi C, Alter H, Mair C. Co-Occurrence of Multiple Risk Factors and Intimate Partner Violence in an Urban Emergency Department. *West J Emerg Med*. 2020;21(2):282-290.
48. Caetano R, Cunradi CB, Alter HJ, Mair C, Yau RK. Drinking and Intimate Partner Violence Severity Levels Among U.S. Ethnic Groups in an Urban Emergency Department. Gerson LW, ed. *Acad Emerg Med*. 2019;26(8):897-907.
49. Hankin A, Smith LS, Daugherty J, Houry D. Correlation Between Intimate Partner Violence Victimization and Risk of Substance Abuse and Depression among African-American Women in an Urban Emergency Department. *West J Emerg Med*. 2010;11(3):252-256.
50. Johnson C, Gorchynski J. Intimate partner violence among men presenting to a university emergency department. *Cal J Emerg Med*. 2004;5(2):40-44.
51. Ramchand R, Marshall GN, Schell TL, et al. Alcohol Abuse and Illegal Drug Use Among Los Angeles County Trauma Patients: Prevalence and Evaluation of Single Item Screener. *J Trauma*. 2009;66(5):1461-1467.
52. Sandoval E, Smith S, Walter J, et al. A Comparison of Frequent and Infrequent Visitors to an Urban Emergency Department. *J Emerg Med*. 2010;38(2):115-121.
53. Singh V, Epstein-Ngo Q, Cunningham RM, Stoddard SA, Chermack ST, Walton MA. Physical dating violence among adolescents and young adults with alcohol misuse. *Drug Alcohol Depend*. 2015;153:364-368.
54. Singh V, Walton MA, Whiteside LK, et al. Dating Violence Among Male and Female Youth Seeking Emergency Department Care. *Ann Emerg Med*. 2014;64(4):405-412.e1.
55. Zun LS, Rosen JM. Psychosocial needs of young persons who are victims of interpersonal violence: *Pediatr Emerg Care*. 2003;19(1):15-19.
56. Biros MH. The Prevalence and Perceived Health Consequences of Hunger in Emergency Department Patient Populations. *Acad Emerg Med*. 2005;12(4):310-317.
57. Gattu, Paik, Wang, Ray, Lichenstein, Black. The Hunger Vital Sign Identifies Household Food Insecurity among Children in Emergency Departments and Primary Care. *Children*. 2019;6(10):107.
58. Kersey MA, Beran MS, McGovern PG, Biros MH, Lurie N. The Prevalence and Effects of Hunger in an Emergency Department Patient Population. *Acad Emerg Med*. 1999;6(11):1109-1114.
59. Makelarski JA, Abramsohn E, Benjamin JH, Du S, Lindau ST. Diagnostic Accuracy of Two Food Insecurity Screeners Recommended for Use in Health Care Settings. *Am J Public Health*. 2017;107(11):1812-1817.
60. Pabalan L, Dunn R, Gregori K, et al. Assessment of Food Insecurity in Children’s Hospital of Wisconsin’s Emergency Department. *W*is Med J. 2015;114(4):148-151.
61. Sullivan AF, Clark S, Pallin DJ, Camargo CA. Food Security, Health, and Medication Expenditures of Emergency Department Patients. *J Emerg Med*. 2010;38(4):524-528.
62. Doran KM, Johns E, Schretzman M, et al. Homeless Shelter Entry in the Year After an Emergency Department Visit: Results From a Linked Data Analysis. *Ann Emerg Med*. 2020;76(4):462-467.
63. Doran KM, Vashi AA, Platis S, et al. Navigating the Boundaries of Emergency Department Care: Addressing the Medical and Social Needs of Patients Who Are Homeless. *Am J Public Health*. 2013;103(S2):S355-S360.
64. Feldman BJ, Calogero CG, Elsayed KS, et al. Prevalence of Homelessness in the Emergency Department Setting. *West J Emerg Med*. 2017;18(3):366-372.
65. Feldman BJ, Craen AM, Enyart J, et al. Prevalence of Homelessness by Gender in an Emergency Department Population in Pennsylvania. *J Osteopath Med*. 2018;118(2):85-91.
66. Lee SJ, Thomas P, Newnham H, et al. Homeless status documentation at a metropolitan hospital emergency department. *Emerg Med Australas*. 2019;31(4):639-645.
67. Orenstein JB, Boenning DA, Engh EP, Zimmerman SJ. Emergency care of children in shelters: *Pediatr Emerg Care*. 1992;8(6):313-317.
68. Pilossoph-Gelb S, Mower WR, Ajaelo I, Yang SC. Psychosocial Difficulties and Emergency Department Use. *Acad Emerg Med*. 1997;4(6):589-592.
69. Post LA, Vaca FE, Doran KM, et al. New Media Use by Patients Who Are Homeless: The Potential of mHealth to Build Connectivity. *J Med Internet Res*. 2013;15(9):e195.
70. Salhi BA, White MH, Pitts SR, Wright DW. Homelessness and Emergency Medicine: A Review of the Literature. Mitchell AM, ed. *Acad Emerg Med*. 2018;25(5):577-593.
71. Carson SM. Implementation of a Comprehensive Program to Improve Child Physical Abuse Screening and Detection in the Emergency Department. *J Emerg Nurs*. 2018;44(6):576-581.
72. Cunningham RM, Murray R, Walton MA, et al. Prevalence of Past Year Assault Among Inner-City Emergency Department Patients. *Ann Emerg Med*. 2009;53(6):814-823.e15.
73. Eulitt PJ, Tomberg RJ, Cunningham TD, Counselman FL, Palmer RM. Screening Elders in the Emergency Department at Risk for Mistreatment: A Pilot Study. *J Elder Abuse Negl*. 2014;26(4):424-435.
74. Greenbaum VJ, Dodd M, McCracken C. A Short Screening Tool to Identify Victims of Child Sex Trafficking in the Health Care Setting: *Pediatr Emerg Care*. 2018;34(1):33-37.
75. Greenbaum VJ, Livings MS, Lai BS, et al. Evaluation of a Tool to Identify Child Sex Trafficking Victims in Multiple Healthcare Settings. *J Adolesc Health*. 2018;63(6):745-752.
76. Hankin A, Wei S, Foreman J, Houry D. Screening for Violence Risk Factors Identifies Young Adults at Risk for Return Emergency Department Visit for Injury. *West J Emerg Med*. 2014;15(5):609-614.
77. Hexom B, Fernando D, Manini AF, Beattie LK. Survivors of Torture: Prevalence in an Urban Emergency Department: SURVIVORS OF TORTURE: PREVALENCE IN AN URBAN ED. *Acad Emerg Med*. 2012;19(10):1158-1165.
78. Kaltiso SO, Greenbaum VJ, Agarwal M, et al. Evaluation of a Screening Tool for Child Sex Trafficking Among Patients With High‐Risk Chief Complaints in a Pediatric Emergency Department. Hwang U, ed. *Acad Emerg Med*. 2018;25(11):1193-1203.
79. Mercier É, Nadeau A, Brousseau AA, et al. Elder Abuse in the Out-of-Hospital and Emergency Department Settings: A Scoping Review. *Ann Emerg Med*. 2020;75(2):181-191.
80. Platts-Mills TF, Dayaa JA, Reeve BB, et al. Development of the Emergency Department Senior Abuse Identification (ED Senior AID) tool. *J Elder Abuse Negl*. 2018;30(4):247-270.
81. Platts-Mills TF, Hurka-Richardson K, Shams RB, et al. Multicenter Validation of an Emergency Department–Based Screening Tool to Identify Elder Abuse. *Ann Emerg Med*. 2020;76(3):280-290.
82. Ranney ML, Patena JV, Nugent N, et al. PTSD, cyberbullying and peer violence: prevalence and correlates among adolescent emergency department patients. *Gen Hosp Psychiatry*. 2016;39:32-38.
83. Richmond NL, Zimmerman S, Reeve BB, et al. Ability of Older Adults to Report Elder Abuse: An Emergency Department–Based Cross‐Sectional Study. *J Am Geriatr Soc*. 2020;68(1):170-175.
84. Johnson T, Patel R, Scott N, et al. Access to Disease Treatment Among Patients Presenting to the Emergency Department with Asthma or Hypertension. *J Emerg Med*. 2015;48(5):527-535.
85. Abbott J. Domestic violence against women. Incidence and prevalence in an emergency department population. *JAMA*. 1995;273(22):1763-1767.
86. Anglin D, Sachs C. Preventive Care in the Emergency Department: Screening for Domestic Violence in the Emergency Department. *Acad Emerg Med*. 2003;10(10):1118-1127.
87. Bazargan-Hejazi S, Kim E, Lin J, Ahmadi A, Khamesi MT, Teruya S. Risk Factors Associated with Different Types of Intimate Partner Violence (IPV): An Emergency Department Study. *J Emerg Med*. 2014;47(6):710-720.
88. Brignone L, Gomez AM. Double jeopardy: Predictors of elevated lethality risk among intimate partner violence victims seen in emergency departments. *Prev Med.* 2017;103:20-25.
89. Clark CJ, Wetzel M, Renner LM, Logeais ME. Linking partner violence survivors to supportive services: impact of the M Health Community Network project on healthcare utilization. *BMC Health Serv Res*. 2019;19(1):479.
90. Dearwater SR. Prevalence of Intimate Partner Abuse in Women Treated at Community Hospital Emergency Departments. *JAMA*. 1998;280(5):433.
91. Ernst AA, Nick ‡ TG, Weiss SJ, Houry§ D, Mills T. Domestic Violence in an Inner-City ED. *Ann Emerg Med*. 1997;30(2):190-197.
92. Ernst AA, Weiss SJ, Cham E, Hall L, Nick TG. Detecting Ongoing Intimate Partner Violence in the Emergency Department Using a Simple 4-Question Screen: The OVAT. *Violence Vict*. 2004;19(3):375-384.
93. Ernst AA, Weiss SJ, Morgan-Edwards S, et al. Derivation and Validation of a Short Emergency Department Screening Tool for Perpetrators of Intimate Partner Violence: The PErpetrator RaPid Scale (PERPS). *J Emerg Med*. 2012;42(2):206-217.
94. Feldhaus KM, Koziol-McLain J, Amsbury HL, Norton IM, Lowenstein SR, Abbott JT. Accuracy of 3 brief screening questions for detecting partner violence in the emergency department. *JAMA*. 1997;277(17):1357-1361.
95. Goncy EA, Rothman EF. The Reliability and Validity of the Dating Abuse Perpetration Acts Scale in an Urban, Emergency Department-Based Sample of Male and Female Youth. *J Interpers Violence*. 2019;34(11):2246-2268.
96. Harland KK, Peek-Asa C, Saftlas AF. Intimate Partner Violence and Controlling Behaviors Experienced by Emergency Department Patients: Differences by Sexual Orientation and Gender Identification. *J Interpers Violence*. 2021;36(11-12):NP6125-NP6143.
97. Little KJ. Screening for domestic violence: Identifying, assisting, and empowering adult victims of abuse. *Postgrad Med*. 2000;108(2):135-141.
98. Litzau M, Denise Dowd M, Stallbaumer-Rouyer J, Miller MK, Randell KA. Universal Intimate-Partner Violence Assessment in the Pediatric Emergency Department and Urgent Care Setting: A Retrospective Review. *Pediatr Emerg Care*. 2019;Publish Ahead of Print.
99. Lo Vecchio F, Bhatia A, Sciallo D. Screening for domestic violence in the emergency department. *Eur J Emerg Med*. 1998;5(4):441-444.
100. Mathew A, Smith LS, Marsh B, Houry D. Relationship of Intimate Partner Violence to Health Status, Chronic Disease, and Screening Behaviors. *J Interpers Violence*. 2013;28(12):2581-2592.
101. Mathew AE, Marsh B, Smith LS, Houry D. Association between Intimate Partner Violence and Health Behaviors of Female Emergency Department Patients. *West J Emerg Med*. 2012;13(3):278-282.
102. McCaw B, Berman WH, Syme SL, Hunkeler EF. Beyond screening for domestic violence. *Am J Prev Med*. 2001;21(3):170-176.
103. Mechem CC, Shofer FS, Reinhard SS, Hornig S, Datner E. History of Domestic Violence among Male Patients Presenting to an Urban Emergency Department. *Acad Emerg Med*. 1999;6(8):786-791.
104. Snider C, Webster D, O’Sullivan CS, Campbell J. Intimate Partner Violence: Development of a Brief Risk Assessment for the Emergency Department. *Acad Emerg Med*. 2009;16(11):1208-1216.
105. Wagers B, Gittelman M, Bennett B, Pomerantz W. Prevalence of male adolescent dating violence in the pediatric emergency department. *J Trauma Acute Care Surg*. 2013;75(4):S313-S318.
106. Weiss SJ, Ernst AA, Cham E, Nick TG. Development of a Screen for Ongoing Intimate Partner Violence. *Violence Vict*. 2003;18(2):131-141.
107. Zakrison TL, Rattan R, Milian Valdés D, et al. Universal screening for intimate partner and sexual violence in trauma patients—What about the men? An Eastern Association for the Surgery of Trauma Multicenter Trial. *J Trauma Acute Care Surg*. 2018;85(1):85-90.
108. Zakrison TL, Ruiz X, Gelbard R, et al. Universal screening for intimate partner and sexual violence in trauma patients: An EAST multicenter trial. *J Trauma Acute Care Surg*. 2017;83(1):105-110.
109. Carpenter CR, Shelton E, Fowler S, et al. Risk Factors and Screening Instruments to Predict Adverse Outcomes for Undifferentiated Older Emergency Department Patients: A Systematic Review and Meta-analysis. Wilber ST, ed. *Acad Emerg Med*. 2015;22(1):1-21.
110. Caitlin Thomas-Henkel, Meryl Schulman. *Screening for Social Determinants of Health in Populations with Complex Needs: Implementation Considerations*. Robert Wood Johnson Foundation; 2017. https://www.chcs.org/resource/screening-social-determinants-health-populations-complex-needs-implementation-considerations/
111. Chetty R, Stepner M, Abraham S, et al. The Association Between Income and Life Expectancy in the United States, 2001-2014. *JAMA*. 2016;315(16):1750.
112. Kushel MB, Gupta R, Gee L, Haas JS. Housing instability and food insecurity as barriers to health care among low-income americans. *J Gen Intern Med*. 2006;21(1):71-77.
113. Rodriguez RM, Fortman J, Chee C, Ng V, Poon D. Food, Shelter and Safety Needs Motivating Homeless Persons’ Visits to an Urban Emergency Department. *Ann Emerg Med*. 2009;53(5):598-602.e1.
114. Tang N, Stein J, Hsia RY, Maselli JH, Gonzales R. Trends and Characteristics of US Emergency Department Visits, 1997-2007. *JAMA*. 2010;304(6):664.
115. King CA, Grupp‐Phelan J, Brent D, et al. Predicting 3‐month risk for adolescent suicide attempts among pediatric emergency department patients. *J Child Psychol Psychiatr*. 2019;60(10):1055-1064.
116. Johnson KN, Raetz A, Harte M, et al. Pediatric trauma patient alcohol screening: A 3year review of screening at a Level I Pediatric Trauma Center using the CRAFFT tool. *J Pediatr Surg*. 2014;49(2):330-332.
117. Johnson-Arbor K, Liebman DL, Carter EM. A survey of residential carbon monoxide detector utilization among Connecticut Emergency Department patients. *Clin Toxicol*. 2012;50(5):384-389.
118. Kreuter MW, Thompson T, McQueen A, Garg R. Addressing Social Needs in Health Care Settings: Evidence, Challenges, and Opportunities for Public Health. *Annu Rev Public Health*. 2021;42(1):329-344.
119. Mazer M, Bisgaier J, Dailey E, et al. Risk for Cost-related Medication Nonadherence Among Emergency Department Patients: RISK FOR COST-RELATED MEDICATION NONADHERENCE. *Acad Emerg Med*. 2011;18(3):267-272.
120. Sutcliffe K, Kilgore PE, DeHoff K, et al. Survey of vaccination knowledge and acceptance among adults admitted to an urban emergency department. *Vaccine*. 2017;35(8):1148-1151.
121. Villalona S. Insights from the shadows: exploring deservingness of care in the emergency department and language as a social determinant of health. *Med Humanit*. 2021;47(3):e5-e5.
122. Bernstein SL, Cannata M. Nicotine dependence, motivation to quit, and diagnosis in emergency department patients who smoke. *Addict Behav*. 2006;31(2):288-297.
123. Blow FC, Walton MA, Barry KL, et al. Alcohol and drug use among patients presenting to an inner-city emergency department: A latent class analysis. *Addict Behav*. 2011;36(8):793-800.
124. Cherpitel CJ, Borges G. Screening Instruments for Alcohol Problems: A Comparison of Cut Points between Mexican American and Mexican Patients in the Emergency Room. *Subst Use Misuse*. 2000;35(10):1419-1430.
125. Cherpitel CJ. Comparison of screening instruments for alcohol problems between black and white emergency room patients from two regions of the country. *Alcohol Clin Exp Res*. 1997;21(8):1391-1397.
126. Cunradi CB, Lee J, Pagano A, Caetano R, Alter HJ. Gender Differences in Smoking Among an Urban Emergency Department Sample. *Tob Use Insights*. 2019;12:1179173X1987913.
127. Hankin A, Daugherty M, Bethea A, Haley L. The Emergency Department as a prevention site: A demographic analysis of substance use among ED patients. *Drug Alcohol Depend*. 2013;130(1-3):230-233.
128. Harrison C, Hoonpongsimanont W, Anderson C, et al. Readiness to Change and Reasons for Intended Reduction of Alcohol Consumption in Emergency Department versus Trauma Population. *West J Emerg Med*. 2014;15(3):337-344.
129. Horn K, Leontieva L, Williams JM, Furbee PM, Helmkamp JC, Manley WG. Alcohol problems among young adult emergency department patients: Making predictions using routine sociodemographic information. *J Crit Care*. 2002;17(4):212-220.
130. Linder SH, Sexton K. Conceptual models for cumulative risk assessment. *Am J Public Health*. 2011;101 Suppl 1(Suppl 1):S74-S81.
131. Berkowitz SA, Hulberg AC, Hong C, et al. Addressing basic resource needs to improve primary care quality: a community collaboration programme. *BMJ Qual Saf*. 2016;25(3):164-172.
